# Bilateral primary testicular diffuse large B-cell lymphoma: a case report

**DOI:** 10.1093/jscr/rjab431

**Published:** 2021-10-28

**Authors:** Baskara Batista, Ferry Safriadi

**Affiliations:** Department of Urology, Faculty of Medicine, Universitas Padjadjaran, Hasan Sadikin General Hospital, Bandung, Indonesia; Department of Urology, Faculty of Medicine, Universitas Padjadjaran, Hasan Sadikin General Hospital, Bandung, Indonesia

## Abstract

Primary testicular lymphoma is a rare but aggressive form of extranodal lymphoma.

A 48-year-old man came with painless lump on both testicles since 10 months ago. Testicular tumour marker revealed increased LDH. Testicular USG revealed semi-solid spongiform mass in the right epididymis and bilateral testicles, suggesting malignancy and minimal bilateral hydroceles. Histopathologic examination revealed diffuse large B-cell type lymphoma.

Orchiectomy alone is not the definite treatment. Chemotherapy was given to increase survival rate. It is important to diagnose the disease with adequate diagnostic work up to achieve better prognosis and early treatment.

## INTRODUCTION

Primary testicular lymphoma (PTL) is a rare but aggressive form of extranodal lymphoma. It accounts for 1–2% of all non-Hodgkin’s lymphomas and 4% of extranodal lymphomas. Most PTL is diffuse large B-cell (DLBLC) type, which has the potential for aggressive clinical behaviour [1]. We present a case of synchronous bilateral DLBLC testicular tumours managed in our centre following SCARE 2020 guideline [2].

## CASE REPORT

A 48-year-old man presented with a lump on both testicles since 10 months prior to admission. The patient neglected it and did not seek any treatment due to lack of pain and the patient thought that the size of the lump was tolerable. The lump grew bigger in the last 3 months.

The patient smoked half a pack of cigarettes per day. Previous history of hypertension, diabetes, heart disease and drug consumption. No familial history of malignancies was found.

Vital signs were within normal limits. Scrotal examination revealed enlarged hard masses affecting both testes and right epididymis, with right mass was larger than the left.

**Figure 1:**
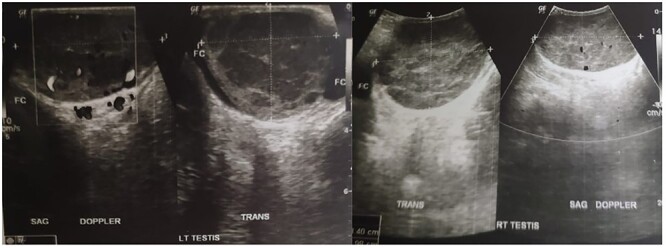
Testicular ultrasonography result.

Complete blood count of the patient was unremarkable. Testicular tumour markers revealed increased in LDH (314 U/L), normal AFP (4,4 ng/mL) and normal bHCG (1,6 mIU/mL). Chest x-ray showed normal result with no lung metastasis. Ultrasonography (USG) examination revealed semi-solid spongiform mass in the right epididymis and bilateral testicles, suggestive malignancy and minimal bilateral hydroceles (Fig. 1). Bilateral radical orchiectomy under general anaesthesia was done.

During surgery, a brownish-white tumour mass was found filling the entire testicular tissue. On the right testicle, there was adhesion of the mass to the surrounding tissue, but it can still be released. Only right spermatic testicular cord appeared infiltrated with mass.

**Figure 2:**
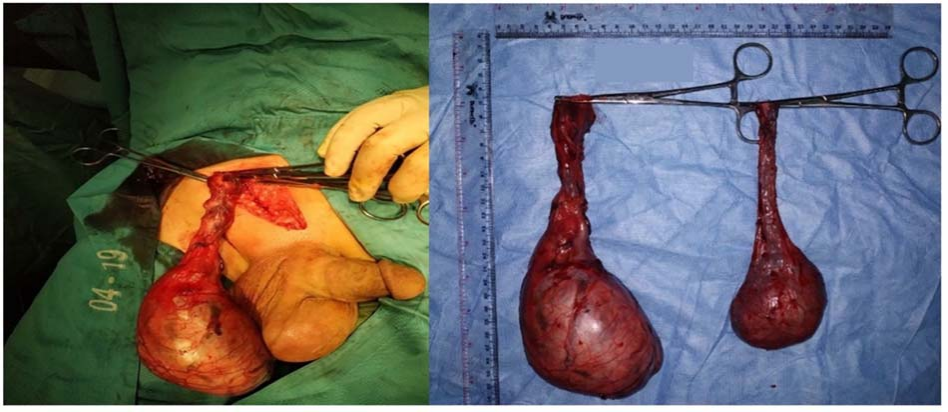
Gross morphology of right and left testicular tumours.

**Figure 3:**
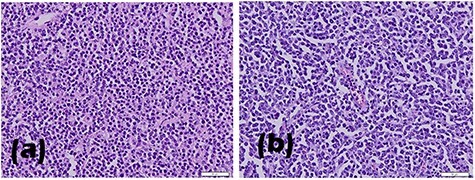
Histopathological results of (a) left and (b) right testicle biopsy.

Macroscopic examination of the excised tumours showed enlarged right testicle with lamellar solid white mass attached to spermatic cord cyst; enlarged left testicle weighed with lobulated mass on lamella (Fig. 2). Histopathological examination revealed infiltration of lympho-vascular invasion of the right and left testicular tumour (Fig. 3). Histopathological reports of both testicles were similar: hyperplastic, dense, pleomorphic, hyperchromatic cells with active mitosis showed bilateral testicular seminoma. Immunohistochemical (IHC) examination of LCA and CD20 showed B Cell as the result, indicated testicles as the primary site of the tumour.

Testicular tumour marker and abdominal pelvic CT scan were performed during follow-up after the surgery to detect any remainder of the malignancy. Compared to pre-surgical result, the testicular tumour marker showed a normal LDH (222 U/L), normal AFP (3,4 ng/mL) and normal bHCG (0,1 mIU/mL). Testicular tumour marker was obtained before histopathological result was available. CT scan result showed non-homogenous lesion with clear borders on the right scrotum (suspected residual mass), no visible residual mass in the left testicle and multiple lymph node enlargement with partial conglomeration in the paraaortic region.

The working diagnosis was bilateral testicular tumour (right testicle grade pT3N2M0S1 and left testicle tumour grade pT2N2M0S1). Right tumour invaded the spermatic cord, and the left was limited to the testis and epididymis. Both tumours invaded surrounding lympho-vascular system. No metastasis was found.

Poly-chemotherapeutic treatment was initiated: six cycles of Rituximab, Cyclophosphamide, Doxorubicin and Vincristine. There was no mass nor metastasis found after the treatment, confirmed by whole abdominal USG in June 2020. The patient was included in the intermediate risk group (increased LDH of 313 U/L, stage III/IV disease) with *International Prognostic Index* (IPI) score of 2. Written informed consent was obtained from the patient for this case report.

## DISCUSSION

PTL usually presents as unilateral [3], firm and painless testicular mass [4], and this could be why the patient in this case neglected his condition. Bilateral synchronous involvement, as seen in this case, occurs only 6–10% of all cases [5]. USG result may show hypoechogenicity with hypervascularity in a diffuse or focal area [6].

Diagnosis is based on history taking, physical examination and USG imaging. HIV serology should better be performed as it has high concurrency with Non-Hodgkin Lymphoma [7]. In this case, testicles revealed enlarged right epididymis and bilateral testicles with minimal bilateral hydrocele, accompanied by spongiform semi-solid lesions in the right epididymis and bilateral testicles; suggestive malignancy according to USG examination. When PTL is suspected, inguinal orchiectomy is required to achieve optimal disease control and adequate pathologic specimen. IHC examination from the case indicated CD20, which strongly positive and diffuse in tumour cells [8].

Blood–testis barrier makes testicular tumours inaccessible to systemic chemotherapy, but it can be removed by orchiectomy. However, even in stage I disease, orchiectomy alone is not a definite treatment as in testicular DLBCL the risk of contralateral testicular relapse is high, 42% at 15 years among patients who did not receive prophylactic radiation in the IELSG study [3, 9]. Prophylactic radiation to contralateral testicle prevented testicular relapse and was also associated with better survival. Similar findings are also seen in other studies [9].

The patient was given Rituximab, Cyclophosphamide, Doxorubicin and Vincristine as chemotherapy agents. Beside Rituximab, other common combination are cyclophosphamide, vincristine, doxorubicin and dexamethasone [10]. Methotrexate is considered an alternative as it shows better impact on CNS than Rituximab [11]. Intrathecal administration of chemotherapy can also help preventing CNS relapse [12].

Studies reported that PTL is a rare malignancy with poor outcome [13]. Ann-Arbor staging system and IPI score can predict the prognosis of Hodgkin’s and non-Hodgkin lymphoma. The prognosis of extensive lymphoma is poor, especially stage III/IV Ann-Arbor and IPI score of ≥2 [14] as seen in this case. As his last appointment occurred in 2020, this patient should do the follow-up management to see any changes as he has poor prognosis and because PTL tends to spread to extra-nodal site, like CNS, contralateral testis, lung and kidney [15].

In conclusion, it is important to diagnose DLBCL with adequate history taking, physical examination, and imaging modalities to achieve better prognosis with early treatment. Family physician or in primary care should also educate the patient about the early symptoms of tumour.
